# Therapeutic Efficacy of Natural Product ‘C-Phycocyanin’ in Alleviating Streptozotocin-Induced Diabetes via the Inhibition of Glycation Reaction in Rats

**DOI:** 10.3390/ijms232214235

**Published:** 2022-11-17

**Authors:** Arbab Husain, Sultan Alouffi, Afreen Khanam, Rihab Akasha, Alvina Farooqui, Saheem Ahmad

**Affiliations:** 1 Department of Biosciences, Faculty of Sciences, Integral University, Lucknow 226026, India; 2 Department of Biotechnology and Life Sciences, Institute of Biomedical Education and Research, Mangalayatan University, Aligarh 202145, India; 3 Department of Medical Laboratory Sciences, College of Applied Medical Sciences, University of Hail, Hail 2440, Saudi Arabia; 4 Molecular Diagnostic & Personalized Therapeutic Unit, University of Hail, Hail 2440, Saudi Arabia; 5 Department of Bioengineering, Faculty of Engineering, Integral University, Lucknow 226026, India

**Keywords:** type 2 diabetes mellitus (T2DM), advanced glycation end products (AGEs), streptozotocin (STZ), *Plectonema* species, c-phycocyanin (C-PC)

## Abstract

Diabetes is a long-term metabolic disorder characterized by persistently elevated blood sugar levels. Chronic hyperglycemia enhances glucose–protein interactions, leading to the formation of advanced glycation end products (AGEs), which form irreversible cross-links with a wide variety of macromolecules, and accumulate rapidly in the body tissues. Thus, the objective of this study was to assess the therapeutic properties of C-phycocyanin (C-PC) obtained from *Plectonema* species against oxidative stress, glycation, and type 2 diabetes mellitus (T2DM) in a streptozotocin (STZ)-induced diabetic Wistar rat. Forty-five days of C-PC administration decreased levels of triglycerides (TGs), blood glucose, glycosylated hemoglobin, (HbA1c), total cholesterol (TC), low-density lipoprotein cholesterol (LDL-C), liver and kidney function indices, and raised body weight in diabetic rats. C-PC suppressed biochemical glycation markers, as well as serum carboxymethyllysine (CML) and fluorescent AGEs. Additionally, C-PC maintained the redox state by lowering lipid peroxidation and protein-bound carbonyl content (CC), enhancing the activity of high-density lipoprotein cholesterol (HDL-C) and renal antioxidant enzymes, and preserving retinal and renal histopathological characteristics. Thus, we infer that C-PC possesses antidiabetic and antiglycation effects in diabetic rats. C-PC may also act as an antidiabetic and antiglycation agent in vivo that may reduce the risk of secondary diabetic complications.

## 1. Introduction

Diabetes mellitus is a severely debilitating metabolic condition manifested by persistent hyperglycemia [1]. Hyperglycemia is caused by the insufficiency of insulin production due to the pancreas (type 1 diabetes mellitus (T1DM)) or insulin resistance (type 2 diabetes mellitus (T2DM)) [2]. According to the International Diabetes Federation’s (IDF) Diabetes Atlas, the overall population suffering from diabetes between the ages of 20 and 79 comprised 469 million individuals in 2019, which is predicted to increase to 578 million by 2030 [3].

Long-term diabetes mellitus is characterized by its vital complications, including retinopathy and nephropathy, etc. Retinopathy may result in visual loss, nephropathy can lead to kidney failure, while peripheral neuropathy is associated with Charcot’s joint amputations and the risk of foot ulcers [4]. There are more cardiovascular, peripheral artery, and cerebrovascular disease cases in patients with diabetes [5].

In diabetes, consistent high glucose levels may induce glycation, which leads to advanced glycation end product (AGE) formation and, consequently, generates various diabetic problems [6]. Glycation is a nonenzymatic process that occurs when a reducing sugar’s carbonyl group reacts with nucleic acids, lipids, and the amino group of proteins [7,8]. Albumin, fibrinogen, and globulin are plasma proteins that can be modified nonenzymatically, and these modifications can have numerous negative effects, including altered drug affinity in the plasma, platelet activation, the production of oxygen free radicals, impaired fibrinolysis, and immune system modulation [9,10]. Glycation plays a significant role in the development and progression of diabetic complications such as retinopathy, nephropathy, and neuropathy [11,12].

Presently, the basic management strategy for diabetes mellitus is to maintain and reduce blood sugar levels in the vessels to normal levels [13]. Worldwide, there are six major classes of modern medications used to treat hyperglycemia and two types of injections [14]. Glitazones, metformin, glinides, dipeptidyl peptidase-4 inhibitors, sulfonylureas, and alpha-glucosidase inhibitors are the generic names for the medications. Insulin and incretin mimetics are a class of injectable medicines. Most modern medicines, on the other hand, have numerous side effects that can result in serious medical issues over time, including a twofold increased risk of pancreatitis, congestive heart failure, increased low-density lipoprotein cholesterol (LDL-C), ketoacidosis, bone fractures, lipoatrophy, lipohypertrophy, skin allergy, hypoglycemia, gastrointestinal upset, skin rash, edema, fluid retention, weight gain, nausea, lactic acidosis, liver function impairment, diarrhea, and flatulence, etc. [15,16].

In addition to modern medicine, traditional medicine has been practiced for a long time, and continues to play a significant role as an alternative medicine [17]. Mostly in underdeveloped nations, plant-based traditional systems, according to WHO, are still the primary source of nutrition for roughly 75–80% of the world’s population [18]. Numerous patients with T2DM have benefited from functional foods such as turmeric, fenugreek, and olive leaf extract, to lower their blood sugar levels [19]. Therefore, the need to discover more effective, safe, cost-manageable, and innovative antidiabetic medications with limited adverse effects is critical in clinical research [14].

Natural products are becoming more important in the research and development of new medications. Along a wide range of species, several natural substances have been recognized and assessed for their medicinal potential. These active components of plant origin have provided some vital molecules for drug designing. The search for natural products has changed drug development procedures [20]. Cyanobacteria are known for their widespread abundance. Cyanobacteria are thought to be promising components for agriculture, food processing, and pharmaceuticals [21]. Cyanobacteria have been shown to have antiviral, antibacterial, algaecide, antifungal, and cytotoxic properties [22,23]. They are said to be rich in natural molecules, such as phenolic compounds, carbohydrates, proteins (phycobiliproteins), as well as pigments. In our preceding study, screening of eight cyanobacterial strains was conducted to evaluate the highest C-phycocyanin (C-PC)-producing strain, and *Plectonema* sp. was identified to be the greatest C-PC producer; in order to isolate, purify, and characterize phycobiliproteins (PBPs), including C-PC, this strain has been utilized further. C-PC has a characteristic light blue color. Depending on the type, it absorbs orange and red light, especially near 620 nm, and emits fluorescence at about 650 nm (also depending on type). Allophycocyanin absorbs and emits light at longer wavelengths than C-phycocyanin or R-phycocyanin. In PBP, the two subunits, i.e., α and β polypeptide chains, are present in equal amounts. Subunit α has a low molecular weight (MW) of 12–19 kDa, while β is 14–21 kDa. These subunits can assemble in various combinations of the trimer (αβ)3 (about 120 kDa) or hexamer (αβ)6 (about 240 kDa) forms [24,25,26].

C-PC, a blue photosensitive pigment generated from cyanobacteria, has been used as a food coloring in ice cream, soft drinks, gum, and sweets, as well as in cosmetics such as lipstick and eyeliner [24]. Because of its fluorescent features, a small quantity of C-PC can also be employed as a biochemical tracer in immunoassays [27]. Moreover, C-PC has been proven to have varied therapeutic properties [28]. The in vitro antioxidant activity of C-PC (in press) leads us to hypothesize that C-PC may act as an antidiabetic and antiglycating agent in vivo that may reduce the risk of secondary diabetic complications.

## 2. Results

### 2.1. Antidiabetic Activity

#### 2.1.1. Body Weight Assessment

**Figure 1:**
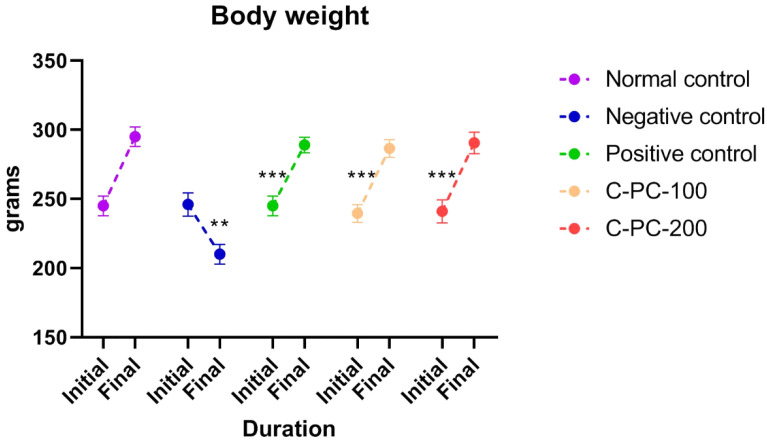
Separated symbols in the graph represent body weight in negative control (n = 5), normal control (n = 5), positive control (n = 5), C-PC-100 (n = 5), and C-PC-200 (n = 5) rats. The data represent the mean plus standard deviation of three different experiments, where ** *p* ≤ 0.01, and *** *p* ≤ 0.001 are regarded as significant.

In the present study, the following groups were used: group I, normal control rats; group II, diabetic control rats (negative control); group III, metformin-treated diabetic rats (positive control); group IV, 100 mg kg^−1^ C-phycocyanin-treated diabetic rats (C-PC-100), and group V, 200 mg kg^−1^ C-phycocyanin-treated diabetic rats (C-PC-200). The initial body weights of all experimental groups showed no obvious difference between the groups. After 45 days of the experiment, the weight of the negative control rats decreased significantly. After the treatment of diabetic rats with C-PC, the body weight of both the C-PC-100 and C-PC-200 (groups IV and V) rats increased significantly, as also observed in the positive control (group III) rats, in comparison to the negative control rats (group II) (Figure 1 and Table 1).

#### 2.1.2. Reduction in the Blood Glucose Levels in C-PC-Treated Rats

**Figure 2:**
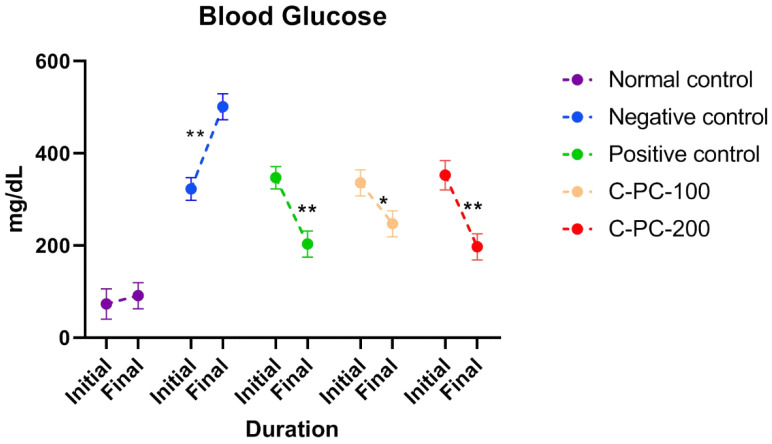
Separated symbols in the graph represent blood glucose levels in normal control, negative control, positive control, C-PC-100, and C-PC-200 rats. The data represent the mean plus standard deviation of three different experiments, where * *p* ≤ 0.05 and ** *p* ≤ 0.01 are regarded as significant.

The diabetes-induced rat groups (II, III, IV, and V) had elevated blood glucose levels on the first day of dosing in comparison to the healthy control rats (group I). Comparing negative control (group II) rats to normal control rats after 45 days of dosing, the negative control (group II) rats had higher blood glucose levels (group I). When diabetic rats were treated with C-PC, blood glucose levels were significantly lower in the C-PC-100 and C-PC-200 (groups IV and V) rats than in the negative control (group II) rats. Additionally, after 45 days of treatment, the blood glucose concentration markedly dropped in the C-PC-200 (group V) rats, as also occurred in the positive control (group III) rats (Figure 2 and Table 1).

#### 2.1.3. Reduction in the HbA1c Levels in C-PC-Treated Rats

**Figure 3:**
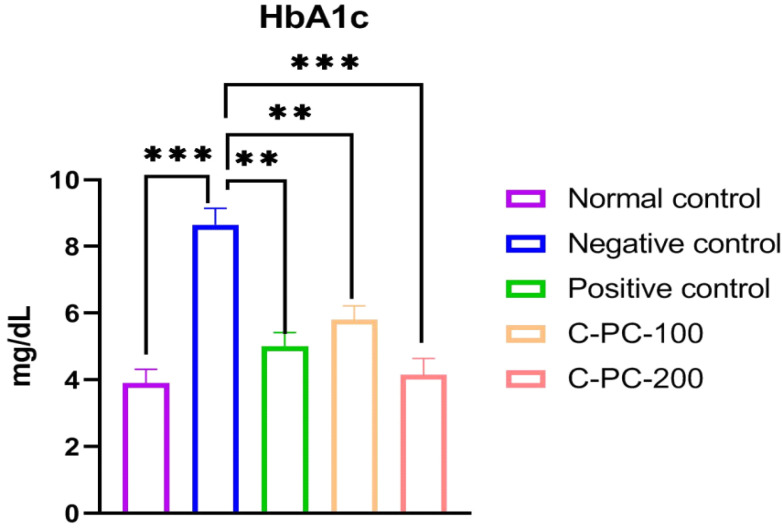
Bar graph representing HbA1c levels in normal control, negative control, positive control, C-PC-100, and C-PC-200 rats. The data represent the mean plus standard deviation of three different experiments, where ** *p* ≤ 0.01, and *** *p* ≤ 0.001 are regarded as significant.

After 45 days of the experiment, the negative control (group II) rats had higher HbA1c levels than the normal control (group I) rats. However, the HbA1c concentrations in the C-PC-100 and C-PC-200 (groups IV and V) rat models were reduced significantly. When compared to negative control rats (group II), positive control (group III) rats exhibited a substantial drop in HbA1c levels after 45 days of treatment (Figure 3 and Table 1).

#### 2.1.4. Alterations in the Serum Lipid Profile

**Figure 4:**
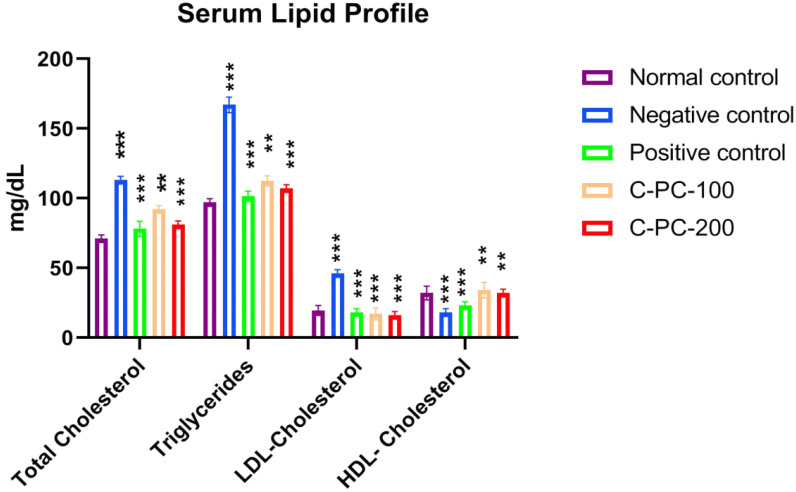
Bar graph representing serum lipid profile in normal control, negative control, positive control, C-PC-100, and C-PC-200 rats. The data represent the mean plus standard deviation of three different experiments, where ** *p* ≤ 0.01, and *** *p* ≤ 0.001 are regarded as significant.

The blood concentrations of TGs, LDL-C, TC, and HDL-C are shown in Figure 4 and Table 1. When compared to the normal control (group I) of rats, the negative control (group II) rats had lower blood concentrations of HDL-C and higher blood levels of TC, LDL-C, and TGs. Positive control, C-PC-100, and C-PC-200 (groups III, IV, and V) rats had considerably lower levels of TC, LDL-C, and TGs than negative control rats (group II); nonetheless, HDL-C levels rose, and were comparable to the reference value (group I).

#### 2.1.5. Alterations in the Liver Function Parameters

**Figure 5:**
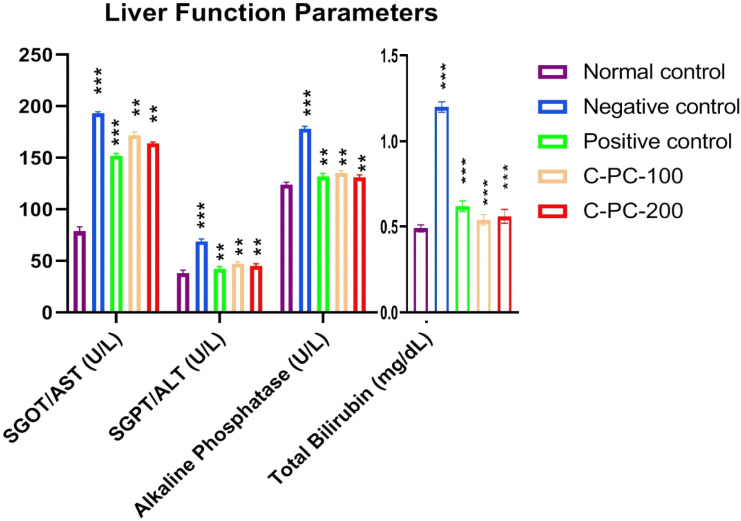
Bar graph representing liver function parameters in normal control, negative control, positive control, C-PC-100, and C-PC-200 rats. The data represent the mean plus standard deviation of three different experiments, where ** *p* ≤ 0.01, and *** *p* ≤ 0.001 are regarded as significant.

The serum SGOT/AST, SGPT/ALT, alkaline phosphatase, and total bilirubin increased after 45 days of the experiment in the negative control (group II) rats compared to the normal control (group I) rats. However, C-PC-100 and C-PC-200 (groups IV and V) rats showed remarkably decreased SGOT, alkaline phosphatase, SGPT, and total bilirubin levels. After 45 days of the dosing, the positive control (group III) rats exhibited significantly reduced SGOT, alkaline phosphatase, SGPT and total bilirubin levels in comparison to the negative control (group II) rats (Figure 5 and Table 1).

#### 2.1.6. Alterations in the Kidney Function Parameters

**Figure 6:**
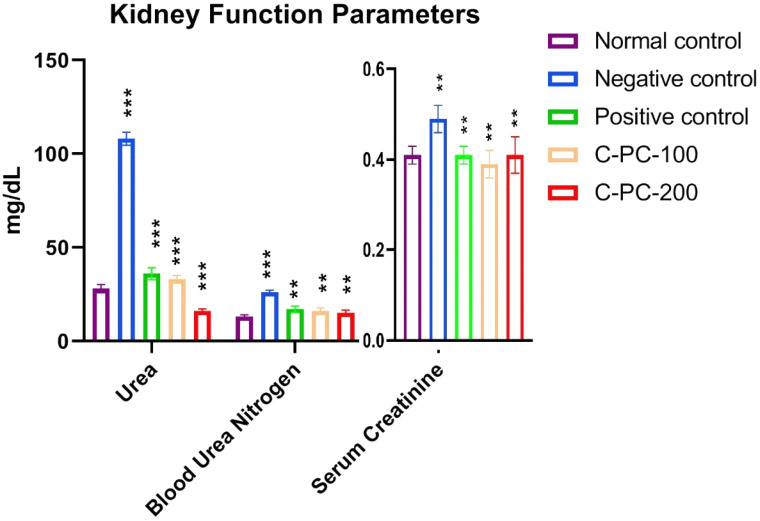
Bar graph represents kidney function parameters in normal control, negative control, positive control, C-PC-100, and C-PC-200 rats. The data represent the mean plus standard deviation of three different experiments, where ** *p* ≤ 0.01, and *** *p* ≤ 0.001 are regarded as significant.

Comparing the negative control (group II) rats to the normal control (group I) rats, a statistically significant rise was seen in all kidney function indices tested. By markedly lowering urea, blood urea nitrogen (BUN), and serum creatinine in diabetic rats, C-PC-100 and C-PC-200 had a favorable effect on the renal function indicators among those rats. However, the C-PC-200 (group V) rats experienced a more prominent beneficial influence (Figure 6 and Table 1).

### 2.2. Antiglycation Parameters

#### 2.2.1. Changes in the Level of Ketoamine

**Figure 7:**
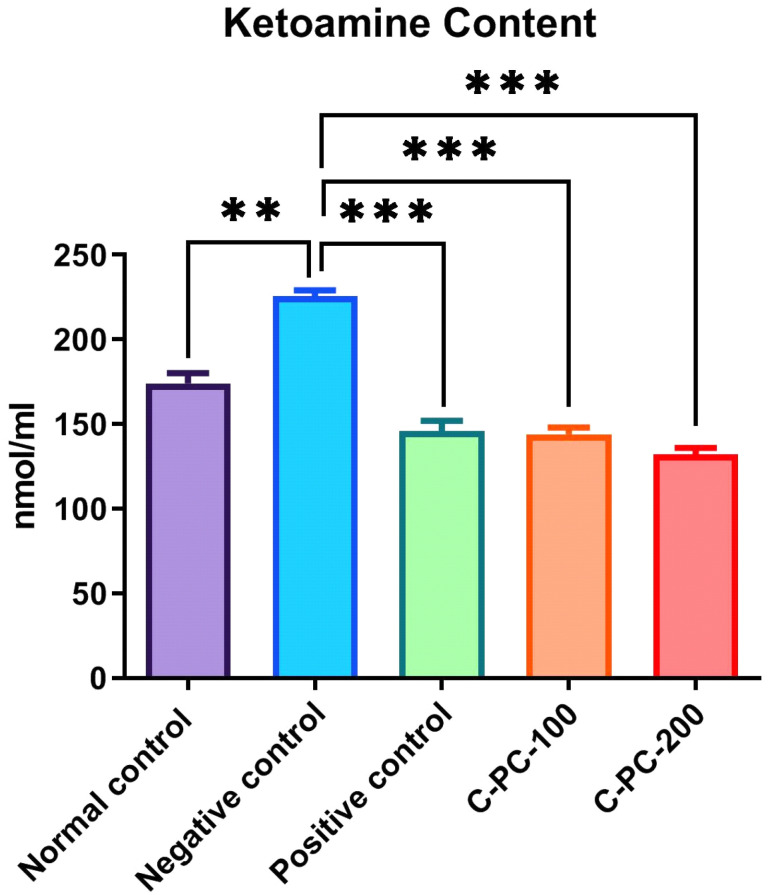
Bar graph representing ketoamine content in normal control, negative control, positive control, C-PC-100, and C-PC-200 rats. The data represent the mean plus standard deviation of three different experiments, where ** *p* ≤ 0.01, and *** *p* ≤ 0.001 are regarded as significant.

To estimate the early glycation adducts in sera, a nitro blue tetrazolium (NBT) assay was performed, which evaluated the inhibitory effect of C-PC by measuring the amount of ketoamines present. Negative control (group II) rats exhibited increased ketoamine content (224 ± 5.1 nmol mL^−1^) compared to that of normal control rats (group I) (132 ± 4.2 nmol mL^−1^). Positive control, C-PC-100, and C-PC-200 (groups III, IV, and V) rats exhibited considerable decreases (143 ± 4.8 nmol mL^−1^, 167.5 ± 3.4 nmol mL^−1^ and 146 ± 5.2 nmol mL^−1^, respectively) in ketoamine content (Figure 7).

#### 2.2.2. Changes in the Amount of Carbonyl Content Moieties

**Figure 8:**
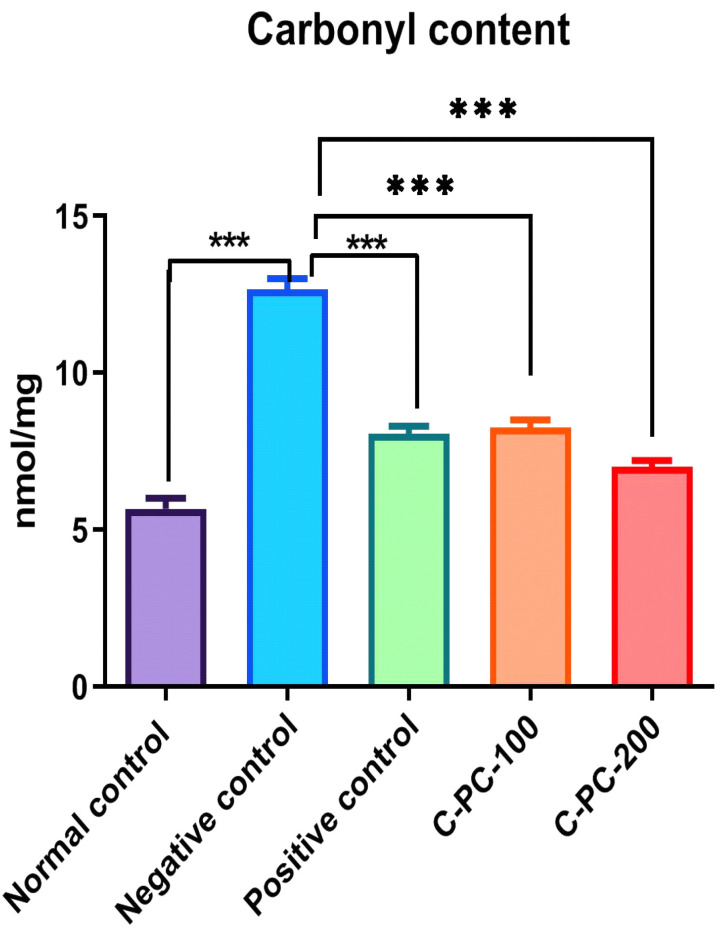
Bar graph representing carbonyl content in normal control, negative control, positive control, C-PC-100, and C-PC-200 rats. The data represent the mean plus standard deviation of three different experiments, where *** *p* ≤ 0.001 is regarded as significant.

An elevation in the protein carbonyl content was recorded in negative control (group II) rats compared to normal control rats (group I). C-PC was able to inhibit the formation of carbonyls in a concentration-dependent manner. Significant inhibitory activities of C-PC were observed in group IV and group V rats. The carbonyl content in groups I, II, III, IV, and V were 5.67 ± 2.3 nmol mg^−1^, 12.65 ± 2.1 nmol mg^−1^, 7.35 ± 1.3 nmol mg^−1^, 8.25 ± 1.2 nmol mg^−1^, and 7.25 ± 0.9 nmol mg^−1^, respectively. The strong inhibitory effect on the carbonyls might be due to the oxidant scavenging property of C-PC (Figure 8).

#### 2.2.3. Detection of GSH

**Figure 9:**
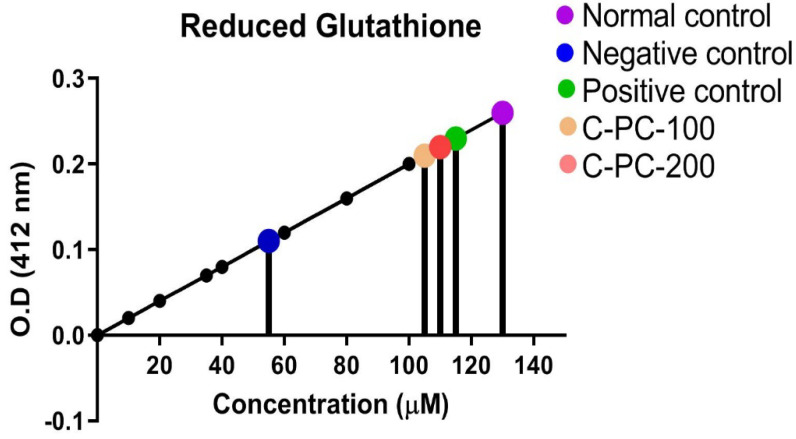
Standard curve for determination of GSH level in normal control, negative control, positive control, C −PC-100, and C −PC −200 rats. The data represent the mean plus standard deviation of three different experiments; highly significance data (*p* ≤ 0.001) are presented.

There was an observable change in the GSH level in the sera of all five groups of rats. In the negative control (group II) rats, GSH level (55 ± 2.8 µM) in the sera was 54% significantly lower as compared to normal control (group I) rats (130 ± 5.1 µM). Furthermore, C-PC-100 (group IV) and C-PC-200 (group V) rats exhibited a statistically significant increase in GSH levels. In the metformin- and C-PC-treated diabetic rats (groups III, IV, and V), sera GSH content was 115 ± 3.10 µM, 105 ± 3.5 µM, and 110 ± 4.10 µM, respectively (Figure 9).

#### 2.2.4. Conjugated Diene and TBARS Estimation

**Figure 10:**
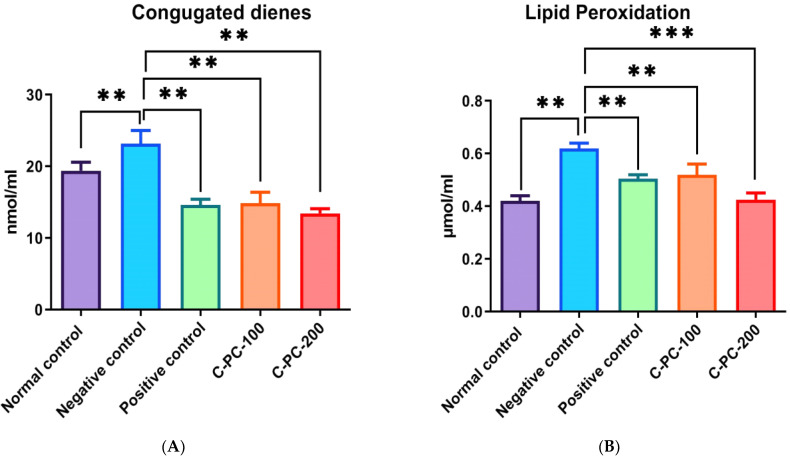
Bar graph representing (**A**) conjugated diene level and (**B**) TBARS level in normal control, negative control, positive control, C-PC-100, and C-PC-200 rats. The data represent the mean plus standard deviation of three different experiments, where ** *p* ≤ 0.01, and *** *p* ≤ 0.001 are regarded as significant.

There was an observable change in the conjugated diene and thiobarbituric acid reactive substances (TBARS) levels in group II, III, IV, and V. In the negative control (group II) rats, conjugated diene and TBARS levels (22.2 ± 2.3 nmol mL^−1^ and 0.62 ± 0.02 µmol mL^−1^, respectively) were statistically significantly higher as compared to normal control (group I) rats (12.3 ± 2.1 nmol mL^−1^ and 0.42 ± 0.03 µmol mL^−1^, respectively). Furthermore, C-PC-100 (group IV) and C-PC-200 (group V) rats showed a significant decrease in conjugated diene and TBARS concentrations. In the positive control (group III), C-PC-100 (group IV), and C-PC-200 (group V) rats, conjugated diene and TBARS levels were 14.8 ± 2.2 nmol mL^−1^, 16.8 ± 2.1 nmol mL^−1^, 14.3 ± 2.3 nmol mL^−1^ and 0.42 ± 0.02 µmol mL^−1^, 0.52 ± 0.03 µmol mL^−1^, 0.43 ± 0.01 µmol mL^−1^, respectively (Figure 10A,B).

##### Carboxymethyllysine (CML) Estimation

**Figure 11:**
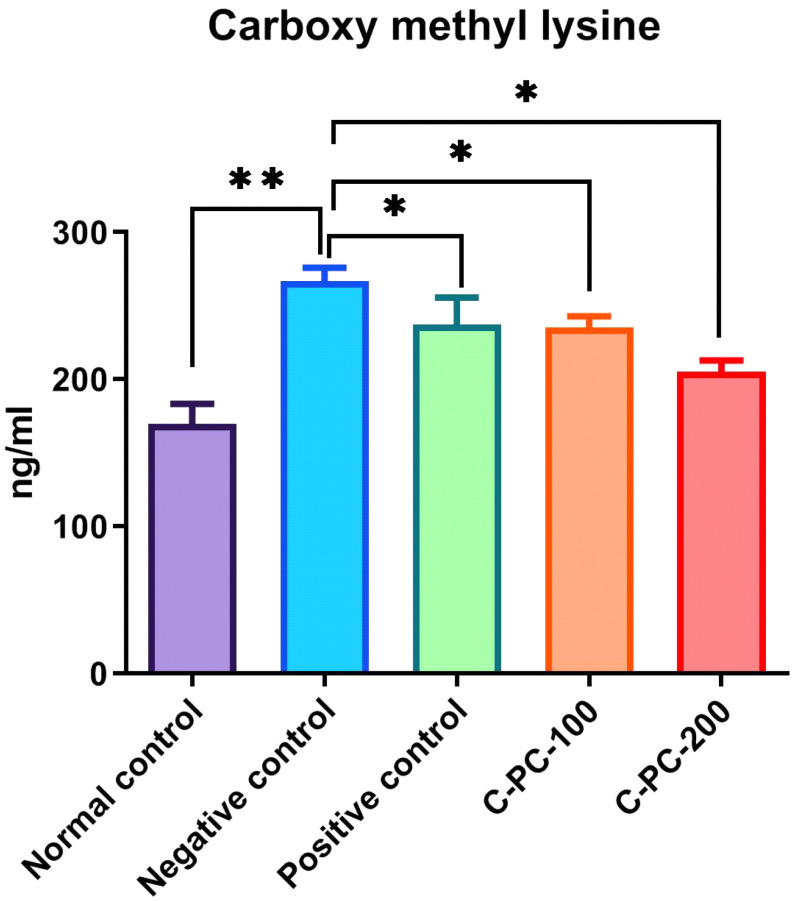
Bar graph representing carboxymethyllysine level in normal control, negative control, positive control, C-PC-100, and C-PC-200 rats. The data represent the mean plus standard deviation of three different experiments, where * *p* ≤ 0.05 and ** *p* ≤ 0.01 are regarded as significant.

The detection of CML in serum samples was performed using a sandwich ELISA kit. The mean content of CML in group I, II, III, IV, and V animal sera samples were 169.7 ± 7.4, 266.8 ± 6.5, 213.4 ± 5.1, 225.5 ± 3.4, and 205.4 ± 5.4 ng/mL, respectively. The sera of negative control (group II) rats showed an increase in the mean contents of CML compared with the sera of normal control (group I) rats, while positive control (group III), C-PC-100 (group IV), and C-PC-200 (group V) rats showed a significant decrease in CML content, as presented in Figure 11.

### 2.3. Histopathological Studies

#### 2.3.1. Kidneys

**Figure 12:**
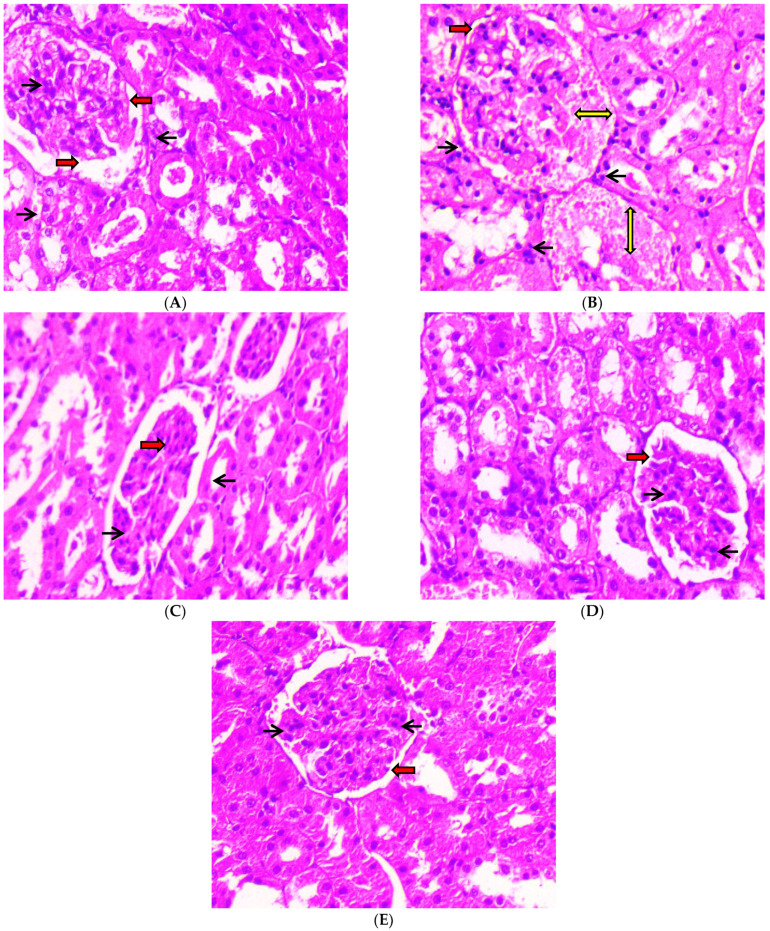
Histopathological study of kidneys (H & E stain) was conducted under a 40× light microscope, after 45 days of administration of C-PC and metformin in STZ-induced diabetic rats. Panel (**A**): Kidney micrograph from a normal control rat showing normal glomerular appearance, normal Bowman’s space (red arrows), and abundant podocytes (black arrows). Panel (**B**): Kidney micrograph from a diabetic (negative control) rat showing abnormal glomerular appearance with glomerulosclerosis (yellow two-sided arrows), enlarged Bowman’s space (red arrows), and reduced podocyte count (black arrows). Panel (**C**): Kidney micrograph from a metformin (positive control)-treated rat showing normal glomerular appearance, normal Bowman’s space (red arrows) and abundant podocytes (black arrows). Panel (**D**): Kidney micrograph from a CP-100-treated rat with slightly normal glomerular appearance, normal Bowman’s space (red arrows), and abundant podocytes (black arrows). Panel (**E**): Kidney micrograph from a C-PC 200-treated rat with slightly normal glomerular appearance, normal Bowman’s space (red arrows), and abundant podocytes (black arrows).

After 45 days of oral administration of 100 mg/kg body weight and 200 mg/kg body weight of C-PC and metformin to STZ-induced diabetic rats, a histopathological investigation of their kidneys was conducted. Photomicrographs of kidneys from normal control (group I) rats revealed typical glomerular lobules and no thickening of the basement membrane, as shown in Figure 12A. The advancement of diffused nodular glomerulosclerosis and capillaries with a thick basement membrane were seen in the photomicrographs of the kidneys from negative control (group II) rats. In contrast, as seen in Figure 12 (B), there were more mesangial cells and a larger Bowman’s space as compared to the kidneys from normal control rats. After 45 days of C-PC treatment, C-PC-200 (group V) rats (Figure 12E) showed a significant reduction in glomerulosclerosis and restoration of the basement membrane; however, kidneys in group IV rats treated with C-PC 100 mg/kg body weight showed only partial restoration to the normal form, with moderate thinning of the glomerular basement membrane (Figure 12D). Additionally, the Bowman’s capsule of positive control (group III) rats was similar to that of the normal control group of rats, and the kidney histology of these rats did not reveal any inflamed blood vessels (Figure 12C).

#### 2.3.2. Eyeballs

**Figure 13:**
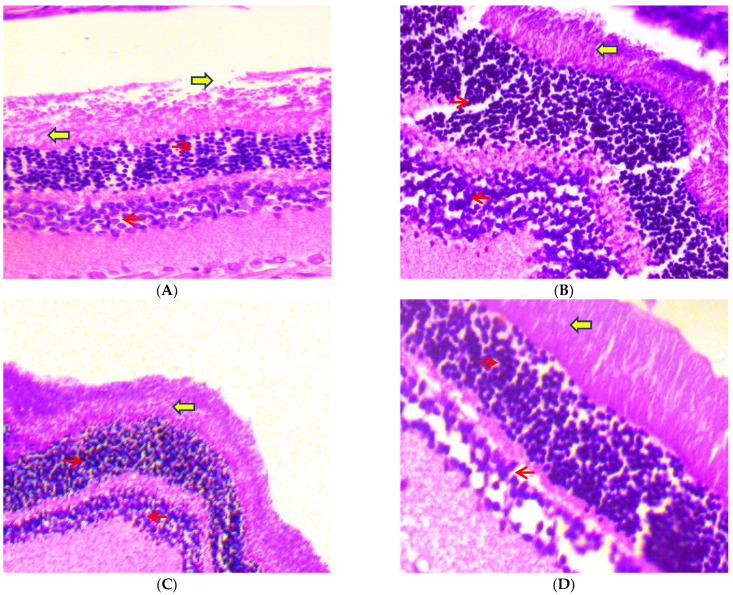

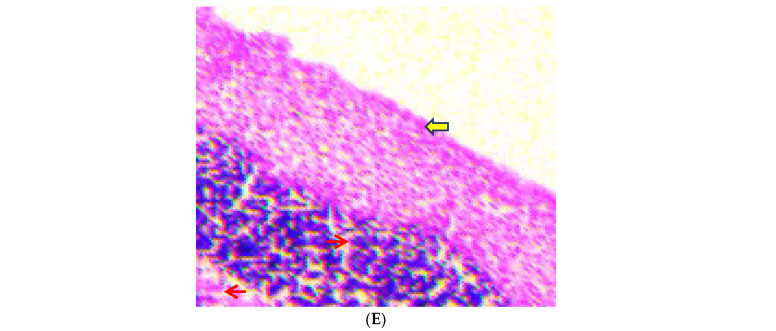
Histopathological study of eyeballs (H & E stain) was conducted under a 40× light microscope, after 45 days of administration of C-PC in STZ-induced diabetic rats. Panel (**A**): Eyeball micrograph from a normal control rat showing normal epithelium (red arrows) and normal stroma (yellow arrows). Panel (**B**): Eyeball micrograph from a diabetic (negative control) rat showing cytoplasmic vacuolations of the epithelial cells and separation of collagen fibrils due to rupturing of the stroma. Panel (**C**): Eyeball micrograph from a metformin (positive control)-treated rat showing that the eyeball architecture was well preserved. Panel (**D**): Eyeball micrograph from a CP-100-treated rat showing increased vascularity along with the slightly thickened basement membrane. Panel (**E**): Eyeball micrograph from a C-PC 200-treated rat exhibiting normal epithelium and slight separation of the collagen, after induction of DM, with mild epithelial cell vacuolation.

After 45 days of receiving oral dosages of different concentrations of C-PC in STZ-induced diabetic rats, histopathological analysis of rat eyeballs was performed. As depicted in Figure 13A, the photomicrographs of the eyeballs from normal control (group I) rats represented normal retinal histology and normal stroma, while the negative control (group II) rats showed increased vascularity in the retinal layer and increased endothelial proliferation, with increased basement thickening and separation of collagen fibrils due to rupturing of stroma (Figure 13B). In the positive control (group III), rats showed normal histology of the eyeball, except a slight increase in endothelial proliferation (Figure 13C). C-PC-100 (group IV) rats exhibited increased vascularity along with capillary changes, including endothelial cell proliferation, with a slightly thickened basement membrane (Figure 13D). C-PC-200 (group V) rats showed slightly increased vascularity, having mild endothelial vascularity (Figure 13E).

## 3. Discussion

In diabetes mellitus, hyperglycemia is thought to be the leading cause of micro- and macrovascular complications; if hyperglycemia persists, the concentration of advanced glycation end products (AGEs) rises [25]. The more AGEs there are in the body, the more likely it is that diabetes will become worse [11].

Several antidiabetic drugs, as well as synthetic inhibitors of glycation, are available that may further reduce the development of diabetic complications; however, numerous toxic effects have been reported after using these drugs [26]. Therefore, the discovery of natural antidiabetic drugs that inhibit protein glycation may provide a promising therapeutic approach to prevent complications in diabetes.

Thus, in the present study, we investigated the ability of C-PC to prevent diabetes and inhibit glycation using an in vivo model. For this, we induced diabetes in Wistar rats using streptozotocin (STZ). STZ is a prominent drug used to induce diabetes in rats. This toxin damages pancreatic cells by alkylating DNA and reducing DNA synthesis and insulin release. When rats are administered upwards of 40 mg/kg body weight of STZ, their pancreatic beta-cells are destroyed, and they develop chronic hyperglycemia, which is analogous to diabetes in humans [28]. Over the course of 72 h, rats receiving STZ injections at a dosage of 60 mg/kg body weight had a rise in blood glucose levels, perhaps as a consequence of the destruction of pancreatic islets and the death of β-cells. Diabetes mellitus causes body cells to rely on proteins for energy rather than glucose. As a consequence, protein storage decreases and body weight decreases [29].

The body weight of the STZ-induced diabetic rats (negative control) in this study decreased throughout the experiment. For 45 days, oral administration of C-PC to diabetic rats resulted in a considerable increase in body weight compared to the negative control animals. C-PC-treated rats had significantly lower blood glucose levels than negative control animals. In addition, the blood glucose levels of positive control rats decreased considerably, equivalent to C-PC-200 rats.

The American Diabetes Association has recognized glycated hemoglobin (HbA1c) as a practical alternative to fasting blood glucose in the diagnosis of diabetes [29]. HbA1c is an essential biomarker of long-term glycemic management, since it might represent the whole glycemic history [30]. There is a strong correlation between HbA1c and the risk of long-term diabetes complications. This means that it is an effective way to measure chronic hyperglycemia [31]. STZ-induced diabetes rats had significantly increased concentrations of HbA1c compared to the control group. C-PC treatment substantially lowered HbA1c levels in diabetic rats compared to negative control animals. Additionally, the positive control rats had essentially identical HbA1c levels to the C-PC-200 animals.

Multiple metabolic disorders are usually linked to diabetes, including lipid metabolism disorders. This is because high concentrations of TGs, LDL-C, and TC and low concentrations of HDL-C are often found in people with diabetes [19]. In the blood, TC, TG, and LDL-C levels increase while HDL-C levels fall, leading to diabetes-related secondary complications [19]. In comparison to normal control rats, negative control rats had elevated LDL-C, TG, TC, and lower HDL-C concentrations. C-PC treatment resulted in a significantly lowered lipid profile, including TC, TGs, and LDL-C, comparable to the reference level, and a considerably raised HDL-C concentration, consistent with the positive control rats. These findings may be related to the possible protective effect of the C-PC on pancreatic β-cells.

Furthermore, C-PC also possesses a protective effect on liver function parameters such as SGOT, alkaline phosphatase, SGPT, and total bilirubin. Treatment of diabetic rats with C-PC significantly decreased the SGOT, alkaline phosphatase, SGPT, and total bilirubin levels. Similar results were obtained from positive control rats. The reduction in the activity of these enzymes in diabetic rats treated with C-PC suggests a hepatoprotective function in preventing diabetes complications. Similar results were also obtained from kidney function parameters, where C-PC significantly reduced the level of urea, BUN, and serum creatinine in comparison to the negative control rats. Positive control rats also showed decreased levels of kidney function parameters.

During long-term hyperglycemic states associated with diabetes mellitus, glucose forms nonenzymatic adducts with plasma proteins, a process known as glycation. Pathogenesis of diabetes complications is aided by protein glycation and the production of advanced glycation end products (AGEs) [32]. Firstly, to validate the role of C-PC as an antiglycating agent, an NBT assay was performed. The reaction with NBT is a well-established technique for detecting ketoamines, which are a hallmark of early glycation products [33]. The ketoamine or Amadori moieties produced as a result of glycation were determined using the NBT assay [34]. The ketoamine content was found to be highest in diabetic rats (negative control). However, upon treatment with C-PC, ketoamine content decreased close to that of positive control rats. Additionally, protein carbonyl content is one of the reliable biomarkers of the glycation reaction [35]. Hence, in the assessment of the oxidative biomarkers, we found maximum protein carbonyl contents in the negative control rats and minimum in the C-PC-200 rats. Meanwhile, positive control rats and C-PC-100 rats have approximately the same carbonyl contents. Hyperglycemia causes the generation of free radicals, which impairs the body’s defense systems, resulting in cellular dysfunction, membrane oxidative damage, and an higher vulnerability to lipid peroxidation [36]. GSH is an important enzymatic antioxidant that could help prevent lipid peroxidation, and is implicated in cellular defense systems, protecting tissues from oxidative damage [37]. In hyperglycemic conditions, antioxidants are considered to restore damaged extracellular matrix proteins and cell development. Furthermore, hyperglycemic rats have been shown to have an increased pancreatic MDA content [38]. As a result, greater MDA stimulation is linked to increased blood glucose levels and the elimination of reactive oxygen species (ROS). Glucose might accelerate the generation of ROS by auto-oxidation and nonenzymatic glycation of proteins [39]. In hyperglycemia, oxidative breakdown of fructosamines may also participate to oxidative stress [40]. In our investigation, we discovered a statistically significant rise in MDA levels and a substantial decrease in GSH levels in the serum of negative control rats. The administration of C-PC to rats, on the other hand, reduced MDA levels and restored GSH activity to a level that was close to normal. The increased activity of GSH shown in this research might contribute to the potential C-PC influence on the scavenging of oxygen free radicals, therefore protecting pancreatic β-cells from the oxidative damage caused by STZ. Conjugated dienes are oxidative stress indicators and markers of glycation [41,42]. Conjugate diene level was found to be maximum in negative control rats, while it was minimum in C-PC-200 rats. Glycation in vivo supports the formation of more permanent, irreversible fluorescent AGEs, known as CML [43]. We detected increased levels of CML in negative control rats. C-PC-treated rats exhibited significantly decreased levels of CML, nearer to those of positive control rats. C-PC-200 rats exhibited a minimum level of CML. Thus, in light of the above findings, it can be concluded that C-PC can act as an effective antiglycating agent in vivo, and thus, it may inhibit further diabetic complications.

In the current examination, the kidneys of negative control rats had a notable advancement of diffused nodular glomerulosclerosis and capillaries with a thick basement membrane. After 45 days, there was also a rise in mesangial cell count and enlargement of the Bowman’s area. Furthermore, the kidneys of C-PC-treated rats revealed considerable recession in glomerulosclerosis and stabilization of the basement membrane. Moreover, the eyeballs of negative control rats showed increased vascularity in the retinal layer and increased endothelial proliferation with increased basement thickening. Meanwhile, the eyeballs of C-PC-treated rats exhibited a small increase in vascularity, minor endothelial vascularity, and slight thickening of the basement membrane.

Combating oxidative stress is one of the therapeutic approaches in the treatment of diabetics. It has been reported that AGE-associated pathology of diabetic complications can be effectively treated using the synergistic action of compounds that provide both antiglycation and antioxidant properties [44]. Thus, in light of the above findings, it can be concluded that C-PC action induces the lowering of plasma glucose and exhibits antioxidant properties. C-PC inhibits the production of free radicals, and thus plays an important role in inhibiting the auto-oxidation of glucose and glycation of proteins, therefore blocking their conversion to AGEs in vivo. Thus, it may prevent the development of further diabetic complications by inhibiting the progression of glycation and oxidative stress.

## 4. Material and Methods

### 4.1. Materials

The materials use in this study are as follows: C-phycocyanin (C-PC) partially purified from *Plectonema* sp. as described previously [26], nitro blue tetrazolium (NBT), 2,4-DNPH, dimethylsulfoxide (DMSO), 9,10-phenanthrenequinone from Qualigens (Mumbai City, India), methanol (HiMedia Laboratories), diethyl ether (Merck, Darmstadt, Germany), formaldehyde (Merck), metformin hydrochloride (Sigma-Aldrich Chemical Company, Burlington, MA, USA), Tris-citrate buffer, one-touch AccuSure glucometer (Microgene Diagnostic Systems Pvt. Ltd., New Delhi, India), methylglyoxal (MG), gluco check strips (Rapid Diagnostics Group of Companies, Delhi, India), malondialdehyde (MDA) from sigma chemical company (USA), aminoguanidine (AG), guanidine hydrochloride, phosphate buffer saline (PBS), carboxymethyllysine (CML) ELISA kit (Bioassay, Hayward, CA, USA), streptozotocin (Sigma-Aldrich). The highest analytical grade chemicals available in the country were used in this study.

### 4.2. Methods

#### 4.2.1. Ethical Statements and Animals

To conduct in vivo study, Wistar rats (male) weighing about 200–250 g and aged 8–10 months were acquired from CSIR-Indian Institute of Toxicology Research, Gheru Campus, Sarojani Nagar Industrial Area, Lucknow-226026, Uttar Pradesh, India. Animals were separated into five cages after acclimation and maintained in a temperature-controlled environment with a 12 h light/dark cycle, ad libitum access to food and water, and treated with care to reduce harm. All methods in this study were carried out in accordance with the rules established by the Committee for the Purpose of Control and Supervision of Experiments on Animals (CPCSEA), New Delhi, India, and approved by the Institutional Animal Ethical Committee (IAEC) of Integral University, Lucknow, India with approval No: IU/IAEC/18/20.

#### 4.2.2. C-PC Acute Toxicity Evaluation

According to Romay et al. (2005) [45], C-PC was dissolved in distilled water and administered to Wistar rats through oral gavage at a dosage of 3 g/kg body weight. Despite the fact that the effective dose range of C-PC in diverse animal models of inflammation is 25 to 300 mg/kg p.o., the C-PC appeared to be rather safe. In comparison to other nonsteroidal anti-inflammatory drugs (NSAIDs), C-PC seems to have minimal toxicity and no side effects in rats and mice. For rats and mice, the observed LD50 values were calculated to be larger than 3 g/kg. Even at the greatest dosage of C-PC tested (3 g/kg p.o.), no mortality was seen. There were no behavioral changes or statistical differences in body weight between treated and untreated animals after 14 days of monitoring [46].

#### 4.2.3. Induction of Diabetes in the Experimental Rats

The rats were randomized and placed into five groups after 1 week of acclimatization, with each group consisting of five Wistar rats. A single intraperitoneal injection of freshly prepared streptozotocin (STZ) (60 mg/kg body weight) in 0.1 M citrate buffer (pH = 4.5) induced type 2 diabetes in overnight fasting rats. Rats were injected with nicotinamide (NA) (120 mg/kg body weight) in normal saline prior to STZ therapy, whereas control rats received normal saline [47]. NA inhibits PARP1 activity and increases NAD+ levels in cells, preventing STZ-induced DNA damage. STZ–NA also causes diabetes that is long-lasting and stable. STZ–NA causes rats to become diabetic, which can be used to study the molecular causes of diabetic complications and test the antidiabetic effects of natural compounds. This is because STZ–NA damages pancreatic β-cells [48,49]. Animals with blood glucose levels of less than 230 mg/dl were not included in the study. After 72 h of STZ administration, rats with signs of polyuria and polydipsia with fasting blood glucose (FBG) of more than 230 mg/dl were selected to be diabetic for further study [50]. A one-touch glucometer was used to check FBG.

#### 4.2.4. Design of Experiments

Each group of five rats (n = 5) was separated into five groups (I–V).

Group I: normal control rats; during this period of time (45 days), rats had unrestricted access to a standard pellet diet.

Group II: diabetic control rats (negative control); for 45 days, diabetic rats had unrestricted access to a standard pellet diet.

Group III: metformin-treated diabetic rats (positive control); a standard pellet diet and metformin treatment (200 mg/kg/day, p.o.) was administered to diabetic rats for 45 days.

Group IV: C-phycocyanin-treated diabetic rats (C-PC-100); for 45 days, diabetic rats were fed a standard pellet diet and treated with C-PC (100 mg/kg/day, p.o.).

Group V: C-phycocyanin-treated diabetic rats (C-PC-200); for 45 days, diabetic rats were fed a standard pellet diet and treated with C-PC (200 mg/kg/day, p.o.).

#### 4.2.5. Preparation of Metformin and C-PC for Dosing

Diabetic group III rats were orally administered a standard antidiabetic homogenized powder form of the drug metformin at a dosage of 200 mg/kg body weight/day, prepared by dissolving in distilled water with vigorous shaking before administration [51,52]. Diabetic rats of groups IV and V received an oral dose of C-PC (100 and 200 mg/kg body weight/day) dissolved in distilled water [53]. The C-PC dosages were selected based on previously published reports [46,54]. The C-PC was administrated to rats orally by intubation daily for 45 days.

#### 4.2.6. Collection of Blood Samples from Experimental Groups of Animals

Blood was collected at the initial and middle stages of the experiment via the retro-orbital plexus, and finally collected by cardiac puncture after 45 days of C-PC administration. Blood samples were collected in sodium fluoride, EDTA, and serum separator collection tubes (SSTs). Plasma and sera were separated in sodium fluoride and SST collection tubes by centrifugation at 2500 rpm for 22 min, respectively. Each group’s separated sera and plasma were pooled, aliquoted, and kept at 4 °C or −20 °C for use in the further estimation of biochemical parameters. Kidney and eyeball samples of control, diabetic, and metformin- and C-PC-treated rats were collected and stored in 10% paraformaldehyde for histopathology studies.

#### 4.2.7. Antidiabetic Activity

##### Body Weight, Blood Glucose, and HbA1c Detection

A one-touch glucometer was used to evaluate blood glucose levels. The HbA1c test was performed with the use of a commercial kit from Bio-Rad [55]. The body weights of rats in all groups were also determined once a week.

##### Lipid Profile, Liver Function, and Kidney Function Parameters

The samples from different animals groups were evaluated for the following parameters: kidney function parameters (blood urea nitrogen (BUN), urea, and serum creatinine), liver function parameters (serum glutamic oxaloacetic transaminase/aspartate aminotransferase (SGOT/AST), serum glutamic pyruvate transaminase/alanine aminotransferase (SGPT/ALT), alkaline phosphatase, and total bilirubin), and serum lipid profile (triglycerides (TGs), low-density lipoprotein cholesterol (LDL-C), total cholesterol (TC), and high-density lipoprotein cholesterol (HDL-C)). Evaluations were conducted in the commercial pathology laboratory, i.e., ALPINE DIAGNOSTIC LABORATORY: A Unit of Zenhance Wellness Pvt. Ltd., Lucknow, India. Clinical chemistry studies were performed on an automated analyzer using standard procedures and diagnostic kits.

#### 4.2.8. In Vivo Glycation Inhibition through C-PC

##### Nitro Blue Tetrazolium (NBT) Assay

To estimate ketoamine moieties in all the experimental rats, an NBT assay was performed. In brief, 20 µL of serum samples and 180 µL of 0.25 M NBT was mixed and placed at 37 °C for 10 min or until a light purple color appeared. Absorbance was taken at 525 nm for NBT spectra analysis. Group I control animal sera served as the control [34,56].

##### Carbonyl Content (CC) Estimation in Animal Sera

First, 400 µL of 2,4-DNPH reagent (10 mM 2,4-DNPH in 2.5 M HCl) and 100 µL of serum from each of the five groups of rat samples were mixed together. subsequently, 0.5 mL of ice-cold 20% trichloroacetic acid (TCA) solution was added after 1 h at room temperature and incubated for 5 min before being centrifuged for 10 min at 1000 rpm and at 4 °C. Using a 1:1 (*v*/*v*) ethanol/ethylacetate solution, the pellet was washed three times. Finally, the pellet was dissolved in 6 M guanidine hydrochloride. The absorbance was measured at 370 nm. Using a 22,000 M^−1^ cm^−1^ extinction coefficient, the results were obtained [57].

##### Detection of Reduced Glutathione (GSH)

All the reagents for GSH determination were prepared as per the protocol previously described with slight modification [58]. In brief, 100 µL of serum from all five groups of rats were mixed in 5 mL test tubes with 0.6 mL of 0.2 M Tris buffer, pH = 8.2, 750 µL of 10 mM 5,5′-dithiobis(2-nitrobenzoic acid) (DTNB). The mixture was brought to 3.0 mL with 1.58 mL of absolute methanol. Sample blanks without DTNB and sample blanks without sample were produced and kept at room temperature for 25 min, with regular shaking, before the color developed 15 min later. UV-visible spectroscopy at 412 nm wavelength detected a rate of 5-thio-2-nitrobenzoic acid production proportional to the total amount of GSH [59].

##### Detection of Conjugated Diene (CD) and Thiobarbituric Acid Reactive Substances (TBARS)

To determine the amount of conjugated diene (CD) in the lipid fraction of serum from all five groups of rats, sera were extracted in chloroform/methanol, 2:1, *v*/*v*, as previously described [60,61]. The organic phases of lipid extracts were dried at room temperature after centrifugation at 1000 rpm for 5 min. The lipid residue was redissolved in cyclohexane after being dried under nitrogen. Using a UV-visible spectrophotometer, the absorbance of lipid extracted CD was determined in nmol mg^−1^ [62]. For the quantitative study of CD concentration, the baseline level of CD was determined from absorbance minima at 234 nm with a molar extinction coefficient of 2.52 × l0^4^ M^−l^ cm^−l^.

TBARS estimation was performed by adding H_2_SO_4_ and phosphotungstic acid. First, 0.1 mL of serum was mixed with 0.4 mL of 0.083 N H_2_SO_4_ and 0.2 mL of 10% phosphotungstic acid. The solutions were centrifuged at 835.15× *g* for 10 min after being kept at room temperature for 5 min. Subsequently, 2 mL of distilled water and 0.5 mL of the TBA reagent—a mixture of 0.37% aqueous TBA, 15% aqueous TCA, and 0.25 N HCl—were added to the pellet mixture. The solutions were heated at 95 °C for 1 h before centrifugation at 3000 rpm for 10 min to obtain the supernatant. At room temperature, the supernatant was allowed to cool. TBARS concentration (μM/mL) was determined using an Eppendorf BioSpectrometer with a molar extinction coefficient of 1.56 × 105 M^−1^ cm^−1^ at 532 nm and a quartz cuvette with a path length of 1 cm. Using standard MDA, the concentration of malondialdehyde (MDA) was measured [63,64,65,66].

##### Carboxymethyllysine (CML) Detection in Animal Sera

CML is a form of nonfluorescent AGE that can be detected with the CML ELISA kit (Bioassay Technology Laboratory). The CML kit detects and quantifies CML protein adducts quickly. All of the samples were diluted 50:100 in phosphate buffer saline (PBS) and coated on 96-well microplates to identify CML. A biotin-conjugated anti-CML antibody was added after a 10 min incubation period at 30 °C. A washing buffer was used to remove the unattached biotin-conjugated anti-CML antibody. Wells coated with biotin-conjugated anti-CML antibody received enzyme streptavidin-HRP before being incubated at 37 °C for 30 min. Coated wells of plates were thoroughly washed with wash buffer to remove the unbound streptavidin-HRP. The quantity of CML determines how much substrate solution is to be added, and color development follows. At 450 nm, the absorbance was measured.

##### Histopathology of the Kidney Tissue Section and Eyeball of Experimental Rats

Control, diabetic, and metformin- and C-PC-treated male Wistar rat tissue sections were preserved in 10% formalin solution, dried with various concentrations of ethanol, cleaned in xylene, and block wax impregnated to observe structural alterations in the kidneys and eyeballs. Kidney paraffin blocks were sliced into 5 μm thick sections, and ocular portions were placed into block holders. The tissue slices were dewaxed with xylene and hydrated with progressively lower grades of alcohol. Sections were stained with eosin (cytoplasmic stain) and hematoxylin (nuclei stain). Once again, the sections were dehydrated with progressively stronger alcohol concentrations before being extracted using xylene, a plasticizer, and xylene mixture (DPX) for microscopic examination. This was conducted prior to final mounting in a combination of distyrene. Hematoxylin and eosin stain was used for light microscopy (H & E staining) [67,68].

### 4.3. Statistical Analysis

The data are shown as mean ± standard deviation (±SD). The statistical significance of the data was assessed using one-way and two-way ANOVA, followed by a Tukey post hoc test using GraphPad Prism (version 5.01), with significance set at * *p* ≤ 0.05, ** *p* ≤ 0.01, and *** *p* ≤ 0.001.

## 5. Conclusions

The present study provides pharmacological insight into the antioxidant, antiglycation, and antidiabetic potential of C-PC. While most of the studies on C-PC are centered on the antioxidant, hypoglycemic, and anti-inflammatory effects, none of the existing studies have focused on the antioxidant and antiglycation potential as a mechanism to manage T2DM. Our results from a previous study (in press) represent the strong antioxidant activity of C-PC in vitro. Accordingly, in this study, C-PC in vivo alleviates the oxidative stress under diabetic conditions through the inhibition of lipid peroxidation, generation of carbonyl content, prevention and/or delay the onset of renal damage, and may also act by blocking the conversion of glycation intermediates to AGEs. The results of the present study demonstrate the control of pathological diabetic conditions in vivo using C-PC as a natural pharmacological agent.

We hope that our study might pave a path for the management of diabetes and its associated complications using the blue-pigment–protein complex C-PC via its potential in the inhibition of oxidation and glycation. Therefore, it may open new avenues for the development of C-PC as a potential natural, oral, and cost-effective hypoglycemic agent. In view of this, C-PC can be introduced for further research as an antioxidant and antiglycation-based antidiabetic compound for clinical trials in T2DM patients.

## Figures and Tables

**Table 1:** The effect of C-PC 100 mg kg^−1^ and C-PC 200 mg kg^−1^ oral administration on biochemical parameters in rats after 45 days. Comparisons between groups I, II, III, IV, and V.

S.No	Parameters	Groups				
		I	II	III	IV	V
		Normal Control	Negative Control	Positive Control	C-PC-100	C-PC-200
1.	Body Weight (grams)					
i	Initial	245 ± 8.4	246 ± 7.5	243 ± 9.1	238 ± 10.2	241 ± 9.7
ii	Final	295 ± 9.4	210 ± 8.9	289 ± 8.2	286 ± 9.5	291 ± 10.1
2.	Blood Glucose (mg/dL)					
i	Initial	73.5 ± 2.51	320.3 ± 2.23	347.8 ± 2.89	335.4 ± 2.45	345.7 ± 2.71
ii	Final	91.7 ± 2.19	501.2 ± 2.31	203.7 ± 2.54	247.9 ± 2.43	197.5 ± 2.61
3.	HbA1c (mg/dL)	3.9 ± 0.7	7.6 ± 1.1	5.0 ± 0.9	5.8 ± 1.2	4.1 ± 1.3
4.	Serum Lipid Profile (mg/dL)					
i	Total Cholesterol	71 ± 1.4	113 ± 2.1	78 ± 2.3	92 ± 1.5	81 ± 1.4
ii	Triglycerides	97 ± 1.5	167 ± 1.8	101 ± 1.4	112 ± 2.0	107 ± 2.2
iii	LDL Cholesterol	20 ± 0.9	46 ± 1.7	18 ± 1.9	17 ± 1.3	16 ± 1.8
iv	HDL Cholesterol	32 ± 1.6	18 ± 1.5	23 ± 1.6	34 ± 1.9	32 ± 1.4
5.	Liver Function Parameters					
i	SGOT/AST (U/L)	79 ± 1.5	193 ± 1.7	152 ± 2.1	172 ± 3.1	164 ± 2.5
ii	SGPT/ALT (U/L)	38 ± 1.8	69 ± 2.4	42 ± 2.3	47 ± 2.1	45 ± 2.2
iii	Alkaline Phosphatase (U/L)	124 ± 2.3	178 ± 2.5	132 ± 2.8	135 ± 2.7	131 ± 2.9
iv	Total Bilirubin (mg/dL)	0.49 ± 0.02	1.2 ± 0.03	0.54 ± 0.2	0.62 ± 0.02	0.56 ± 0.4
6.	Kidney Function Parameters (mg/dL)					
i	Urea	28 ± 2.2	108 ± 3.5	36 ± 3.2	33 ± 2.1	16 ± 1.2
ii	Blood Urea Nitrogen	13 ± 1.1	26 ± 1.2	17 ± 1.7	16 ± 1.8	15 ± 1.5
iii	Serum Creatinine	0.41 ± 0.02	0.49 ± 0.03	0.41 ± 0.02	0.39 ± 0.03	0.41 ± 0.02

## Data Availability

Not applicable.

## References

[1] Ashcroft F.M., Rohm M., Clark A., Brereton M.F. (2017). Is Type 2 Diabetes a Glycogen Storage Disease of Pancreatic β Cells?. Cell Metab..

[2] American Diabetes Association (2018). Classification and Diagnosis of Diabetes: Standards of Medical Care in Diabetesd 2018. Diabetes Care.

[3] International Diabetes Federation (9th Editio). https://diabetesatlas.org/atlas/ninth-edition/.

[4] American Diabetes Association (2013). Standards of Medical Care in Diabetes—2013. Diabetes Care.

[5] Petrie J., Guzik T.J., Touyz R.M. (2018). Diabetes, Hypertension, and Cardiovascular Disease: Clinical Insights and Vascular Mechanisms. Can. J. Cardiol..

[6] Fishman S.L., Sonmez H., Basman C., Singh V., Poretsky L. (2018). The role of advanced glycation end-products in the development of coronary artery disease in patients with and without diabetes mellitus: A review. Mol. Med..

[7] Katakami N. (2018). Mechanism of Development of Atherosclerosis and Cardiovascular Disease in Diabetes Mellitus. J. Atheroscler. Thromb..

[8] Siddiqui S.A.A.Z. (2015). Protein Glycation: A Firm Link to Cause Metabolic Disease and their Complications. J. Glycom. Lipidom..

[9] Khanam A., Ahmad S., Husain A., Rehman S., Farooqui A., Yusuf M.A. (2020). Glycation and Antioxidants: Hand in the Glove of Antiglycation and Natural Antioxidants. Curr. Protein Pept. Sci..

[10] Chhipa A.S., Borse S.P., Baksi R., Lalotra S., Nivsarkar M. (2019). Targeting receptors of advanced glycation end products (RAGE): Preventing diabetes induced cancer and diabetic complications. Pathol. Res. Pract..

[11] Ahmed N., Thornalley P. (2007). Advanced glycation endproducts: What is their relevance to diabetic complications?. Diabetes Obes. Metab..

[12] Razzaq P.A., Iftikhar M., Faiz A., Aman F., Ijaz A., Iqbal S., Khalid A., Sarwar S. (2020). A Comprehensive Review on Antidiabetic Properties of Turmeric. Life Sci. J..

[13] Miller E., Shubrook J.H. (2015). Sodium Glucose Co-Transporter 2 Inhibitors in the Treatment of Type 2 Diabetes Mellitus. Osteopath. Fam. Physician.

[14] Chaudhury A., Duvoor C., Reddy Dendi V.S., Kraleti S., Chada A., Ravilla R., Marco A., Shekhawat N.S., Montales M.T., Kuriakose K. (2017). Clinical Review of Antidiabetic Drugs: Implications for Type 2 Diabetes Mellitus Management. Front. Endocrinol..

[15] Rahimi-Madiseh M., Malekpour-Tehrani A., Bahmani M., Rafieian-Kopaei M. (2016). The research and development on the antioxidants in prevention of diabetic complications. Asian Pac. J. Trop. Med..

[16] Osadebe P.O., Odoh E.U., Uzor P.F. (2014). Natural Products as Potential Sources of Antidiabetic Drugs. Br. J. Pharm. Res..

[17] Yakubu O.E., Imo C., Shaibu C., Akighir J., Ameh D.S. (2019). Effects of Ethanolic Leaf and Stem-bark Extracts of Adansonia digitata in Alloxan-induced Diabetic Wistar Rats. J. Pharmacol. Toxicol..

[18] James P., Davis S.P., Ravisankar V., Nazeem P.A., Mathew D. (2017). Novel Antidiabetic Molecules from the Medicinal Plants of Western Ghats of India, Identified Through Wide-Spectrumin SilicoAnalyses. J. Herbs Spices Med. Plants.

[19] Elberry A.A., Harraz F.M., Ghareib S.A., Gabr S.A., Nagy A.A., Abdel-Sattar E. (2015). Methanolic extract of Marrubium vulgare ameliorates hyperglycemia and dyslipidemia in streptozotocin-induced diabetic rats. Int. J. Diabetes Mellit..

[20] Beutler J.A. (2009). Natural Products as a Foundation for Drug Discovery. Curr. Protoc. Pharmacol..

[21] Singh R., Parihar P., Singh M., Bajguz A., Kumar J., Singh S., Singh V.P., Prasad S.M. (2017). Uncovering Potential Applications of Cyanobacteria and Algal Metabolites in Biology, Agriculture and Medicine: Current Status and Future Prospects. Front. Microbiol..

[22] Rastogi R.P., Sinha R.P. (2009). Biotechnological and industrial significance of cyanobacterial secondary metabolites. Biotechnol. Adv..

[23] Husain A., Alouffi S., Khanam A., Akasha R., Khan S., Khan M., Farooqui A., Ahmad S. (2022). Non-Inhibitory Effects of the Potent Antioxidant C-Phycocyanin from Plectonema Sp. on the in Vitro Glycation Reaction. Rev. Rom. Med. Lab..

[24] Yan M., Liu B., Jiao X., Qin S. (2014). Preparation of phycocyanin microcapsules and its properties. Food Bioprod. Process..

[25] Chawla A., Chawla R., Jaggi S. (2016). Microvasular and Macrovascular Complications in Diabetes Mellitus: Distinct or Continuum?. Indian J. Endocrinol. Metab..

[26] Elosta A., Ghous T., Ahmed N. (2012). Natural Products as Anti-glycation Agents: Possible Therapeutic Potential for Diabetic Complications. Curr. Diabetes Rev..

[27] Golson M.L., Maulis M.F., Dunn J., Poffenberger G., Schug J., Kaestner K.H., Gannon M.A. (2014). Activated FoxM1 Attenuates Streptozotocin-Mediated β-Cell Death. Mol. Endocrinol..

[28] Montilla P., Barcos M., Muñoz M., Muñoz-Castañeda J.R., Bujalance I., Túnez I. (2004). Protective effect of Montilla-Moriles appellation red wine on oxidative stress induced by streptozotocin in the rat. J. Nutr. Biochem..

[29] Sherwani S.I., Khan H.A., Ekhzaimy A., Masood A., Sakharkar M.K. (2016). Significance of HbA1c Test in Diagnosis and Prognosis of Diabetic Patients. Biomark. Insights.

[30] Lacy M.E., Gilsanz P., Karter A.J., Quesenberry C.P., Pletcher M.J., Whitmer R.A. (2018). Long-term Glycemic Control and Dementia Risk in Type 1 Diabetes. Diabetes Care.

[31] Lippi G., Targher G. (2010). Glycated hemoglobin (HbA1c): Old dogmas, a new perspective?. Clin. Chem. Lab. Med..

[32] Singh R., Rao H.K., Singh T.G. (2020). Review Article Advanced Glycated End Products (AGES) in Diabetes and Its Complications: An Insight. Plant Arch..

[33] Shahab U., Faisal M., Alatar A.A., Ahmad S. (2018). Impact of wedelolactone in the anti-glycation and anti-diabetic activity in experimental diabetic animals. IUBMB Life.

[34] Ahmad S., Akhter F., Moinuddin, Shahab U., Khan M.S. (2013). Studies on glycation of human low density lipoprotein: A functional insight into physico-chemical analysis. Int. J. Biol. Macromol..

[35] Koike S., Kayama T., Arai M., Horiuchi Y., Kobori A., Miyashita M., Itokawa M., Ogasawara Y. (2015). Characterization of modified proteins in plasma from a subtype of schizophrenia based on carbonyl stress: Protein carbonyl is a possible biomarker of psychiatric disorders. Biochem. Biophys. Res. Commun..

[36] Prasad K., Mantha S.V., Muir A.D., Westcott N.D. (2000). Protective effect of secoisolariciresinol diglucoside against streptozotocin-induced diabetes and its mechanism. Mol. Cell. Biochem..

[37] Singh V.P., Bali A., Singh N., Jaggi A.S. (2014). Advanced Glycation End Products and Diabetic Complications. Korean J. Physiol. Pharmacol..

[38] Li L.Y., Li L.Q., Guo C.H. (2010). Evaluation of in Vitro Antioxidant and Antibacterial Activities of Laminaria Japonica Polysaccharides. J. Med. Plants Res..

[39] Abirami R.G., Kowsalya S. (2013). Antidiabetic Activity of Ulva Fasciata and Its Impact on Carbohydrate Metabol- Ism Enzymes in Alloxan Induced Diabetic Rats. Int. J. Res. Phytochem. Pharmacol..

[40] Sarmah S., Roy A.S. (2022). A review on prevention of glycation of proteins: Potential therapeutic substances to mitigate the severity of diabetes complications. Int. J. Biol. Macromol..

[41] Inouye M., Mio T., Sumino K. (1999). Link between glycation and lipoxidation in red blood cells in diabetes. Clin. Chim. Acta.

[42] Mahdavifard S., Nakhjavani M. (2020). Thiamine pyrophosphate improved vascular complications of diabetes in rats with type 2 diabetes by reducing glycation, oxidative stress, and inflammation markers. Med. J. Islam. Repub. Iran.

[43] Gill V., Kumar V., Singh K., Kumar A., Kim J.-J. (2019). Advanced Glycation End Products (AGEs) May Be a Striking Link Between Modern Diet and Health. Biomolecules.

[44] Ramkissoon J., Mahomoodally M., Ahmed N., Subratty A. (2013). Antioxidant and anti–glycation activities correlates with phenolic composition of tropical medicinal herbs. Asian Pac. J. Trop. Med..

[45] Romay C., Gonzalez R., Ledon N., Remirez D., Rimbau V. (2005). C-Phycocyanin: A Biliprotein with Antioxidant, Anti-Inflammatory and Neuroprotective Effects. Curr. Protein Pept. Sci..

[46] Ou Y., Lin L., Yang X., Pan Q., Cheng X. (2013). Antidiabetic potential of phycocyanin: Effects on KKAy mice. Pharm. Biol..

[47] Kapoor R., Srivastava S., Kakkar P. (2009). Bacopa monnieri modulates antioxidant responses in brain and kidney of diabetic rats. Environ. Toxicol. Pharmacol..

[48] Szkudelski T. (2012). Streptozotocin-Nicotinamide-Induced Diabetes in the Rat. Characteristics of the Experimental Model. Exp. Biol. Med..

[49] Ghasemi A., Khalifi S., Jedi S. (2014). Streptozotocin-nicotinamide-induced rat model of type 2 diabetes (review). Acta Physiol. Hung..

[50] Hashim A., Alvi S.S., Ansari I.A., Salman Khan M. (2019). Phyllanthus Virgatus Forst Extract and It’s Partially Purified Fraction Ameliorates Oxidative Stress and Retino-Nephropathic Architecture in Streptozotocin-Induced Diabetic Rats. Pak. J. Pharm. Sci..

[51] Majd N.E., Azizian H., Tabandeh M.R., Shahriari A. (2019). Effect of Abelmoschus esculentus powder on ovarian histology, expression of apoptotic genes and oxidative stress in diabetic rats fed with high fat diet. Iran. J. Pharm. Res..

[52] Shawky L.M., Morsi A.A., El Bana E., Hanafy S.M. (2020). The Biological Impacts of Sitagliptin on the Pancreas of a Rat Model of Type 2 Diabetes Mellitus: Drug Interactions with Metformin. Biology.

[53] Ou Y., Lin L., Pan Q., Yang X., Cheng X. (2012). Preventive effect of phycocyanin from Spirulina platensis on alloxan-injured mice. Environ. Toxicol. Pharmacol..

[54] Ou Y., Ren Z., Wang J., Yang X. (2016). Phycocyanin ameliorates alloxan-induced diabetes mellitus in mice: Involved in insulin signaling pathway and GK expression. Chem. Biol. Interact..

[55] Tzeng T.-F., Liou S.-S., Chang C.J., Liu I.-M. (2013). The Ethanol Extract of *Zingiber zerumbet* Attenuates Streptozotocin-Induced Diabetic Nephropathy in Rats. Evid.-Based Complement. Altern. Med..

[56] Ghisaidoobe A.B.T., Chung S.J. (2014). Intrinsic Tryptophan Fluorescence in the Detection and Analysis of Proteins: A Focus on Förster Resonance Energy Transfer Techniques. Int. J. Mol. Sci..

[57] Khanam A., Alouffi S., Rehman S., Ansari I.A., Shahab U., Ahmad S. (2021). An in vitro approach to unveil the structural alterations in d-ribose induced glycated fibrinogen. J. Biomol. Struct. Dyn..

[58] Oyenihi O.R., Brooks N.L., Oguntibeju O.O. (2015). Effects of kolaviron on hepatic oxidative stress in streptozotocin induced diabetes. BMC Complement. Altern. Med..

[59] Rahman I., Kode A., Biswas S.K. (2007). Assay for quantitative determination of glutathione and glutathione disulfide levels using enzymatic recycling method. Nat. Protoc..

[60] Folch J., Lees M., Sloane Stanley G.H. (1957). A simple method for the isolation and purification of total lipides from animal tissues. J. Biol. Chem..

[61] Ahotupa M., Ruutu M., Mäntylä E. (1996). Simple Methods of Quantifying Oxidation Products and Antioxidant Potential of Low Density Lipoproteins. Clin. Biochem..

[62] Alvi S.S., Ansari I.A., Ahmad M.K., Iqbal J., Khan M.S. (2017). Lycopene amends LPS induced oxidative stress and hypertriglyceridemia via modulating PCSK-9 expression and Apo-CIII mediated lipoprotein lipase activity. Biomed. Pharmacother..

[63] Ohkawa H., Ohishi N., Yagi K. (1979). Assay for lipid peroxides in animal tissues by thiobarbituric acid reaction. Anal. Biochem..

[64] Vlassara H., Palace M. (2002). Diabetes and advanced glycation endproducts. J. Intern. Med..

[65] Yagi K. (1976). A simple fluorometric assay for lipoperoxide in blood plasma. Biochem. Med..

[66] Khan M.Y., Alouffi S., Ahmad S. (2018). Immunochemical studies on native and glycated LDL—An approach to uncover the structural perturbations. Int. J. Biol. Macromol..

[67] Akhter F., Khan M.S., Singh S., Ahmad S. (2014). An Immunohistochemical Analysis to Validate the Rationale behind the Enhanced Immunogenicity of D-Ribosylated Low Density Lipo-Protein. PLoS ONE.

[68] Chauhan A., Sharma S. (2016). Comments on: Microvascular and Macrovascular Complications in Diabetes Mellitus: Distinct or Continuum?. Indian J. Endocrinol. Metab..

